# Congestive heart failure and comorbidity as determinants of colorectal cancer perioperative outcomes

**DOI:** 10.1007/s13304-021-01086-4

**Published:** 2021-06-11

**Authors:** Cristina Basso, Nicola Gennaro, Matilde Dotto, Eliana Ferroni, Marianna Noale, Francesco Avossa, Elena Schievano, Paola Aceto, Concezione Tommasino, Antonio Crucitti, Raffaele Antonelli Incalzi, Stefano Volpato, Flavia Petrini, Michele Carron, Maria Caterina Pace, Gabriella Bettelli, Fernando Chiumiento, Antonio Corcione, Marco Montorsi, Marco Trabucchi, Stefania Maggi, Maria Chiara Corti

**Affiliations:** 1 Epidemiological Department (SER), Azienda Zero, Via Jacopo Avanzo 35, Veneto Region, 35132 Padua, Italy; 2 National Research Council (CNR), Neuroscience Institute, Aging Branch, Padua, Italy; 3 Consorzio di Ricerca “Luigi Amaducci”, Padua, Italy; 4 SIAARTI, Italian Society of Anaesthesia, Analgesia, Resuscitation and Intensive Care, Rome, Italy; 5 Fondazione Policlinico Universitario A. Gemelli IRCCS, Rome, Italy; 6 Università Cattolica del Sacro Cuore, Rome, Italy; 7 University of San Marino, San Marino, San Marino; 8 Department of Anaestesia, Intensive Care, Day Surgery and Pain Therapy and Geriatric Surgery Area, IRCCS INRCA, Italian National Research Centres on Aging, Ancona, Italy; 9 Department of Medicine, DIMED, Section of Anesthesiology and Intensive Care, University of Padova, Padua, Italy; 10 Dipartimento Area Critica, ASL Salerno, Salerno, Italy; 11 Dipartimento di Area Critica UOC Anestesia e TIPO, AORN dei Colli-Monaldi, Naples, Italy; 12 Perioperative Medicine, Pain Therapy, ICU and Emergency Department, Chieti-Pescara University, Pescara, Italy; 13 Department of Biomedical, Surgical and Odontoiatric Sciences, University of Milano, Anaesthesia and Intensive Care, Polo Universitario Ospedale San Paolo, Milan, Italy; 14 SICG, Società Italiana di Chirurgia Geriatrica, Naples, Italy; 15 Cristo Re Hospital, Catholic University Rome, Rome, Italy; 16 SIC, Società Italiana di Chirurgia, Rome, Italy; 17 Humanitas University and Research Hospital IRCCS, Milan, Italy; 18 SIGG, Società Italiana di Geriatria e Gerontologia, Florence, Italy; 19 AIP, Società Italiana di Psicogeriatria, Brescia, Italy; 20 Cattedra di Medicina Interna e Geriatria, Università Campus Bio-Medico, Rome, Italy; 21 Dipartimento di Scienze Mediche, Università di Ferrara, Ferrara, Italy; 22 Dipartimento di Scienze Biomediche, Chirurgiche ed Odontostomatologiche, Università di Milano, Milan, Italy

**Keywords:** Age, Comorbidities, Colorectal cancer surgery, Perioperative outcomes, ACG, LOS

## Abstract

There has been an increase in surgical interventions in frailer elderly with concomitant chronic diseases. The purpose of this paper was to evaluate the impact of aging and comorbidities on outcomes in patients who underwent surgery for the treatment of colorectal cancer (CRC) in Veneto Region (Northeastern Italy). This is a retrospective cohort study in patients ≥ 40 years who underwent elective or urgent CRC surgical resection between January 2013 and December 2015. Independent variables included: age, sex, and comorbidities. We analyzed variables associated with the surgical procedure, such as stoma creation, hospitalization during the year before the index surgery, the surgical approach used, the American Society of Anesthesiologists (ASA) score, and the Charlson Comorbidity Index score. Eight thousand four hundred and forty-seven patients with CRC underwent surgical resection. Patient age affected both pre- and post-resection LOS as well as the overall survival (OS); however, it did not affect the 30-day readmission and reoperation rates. Multivariate analysis showed that age represented a risk factor for longer preoperative and postoperative LOS as well as for 30-day and 365-day mortality, but it was not associated with an increased risk of 30-day reoperation and 30-day readmission. Chronic Heart Failure increased the 30-day mortality risk by four times, the preoperative LOS by 51%, and the postoperative LOS by 33%. Chronic renal failure was associated with a 74% higher 30-day readmission rate. Advanced age and comorbidities require a careful preoperative evaluation and appropriate perioperative management to improve surgical outcomes in older patients undergoing elective or urgent CRC resection.

## Introduction

Colorectal cancer (CRC) is the third most common cancer in men and the second most common in women worldwide. It is the second most common cancer in Italy [1–3]. Recent studies have revealed a concomitant rise in CRC incidence rate with age in developed countries. Thus, with longer life expectancies and an aging population, the number of patients with CRC is expected to rise. In addition, based on recent advancements in anesthetic and surgical techniques, the number of older patients undergoing surgery is also expected to rise [4].

In recent decades, there has been a gradual decline in the CRC-related mortality rates [1–3, 5]; however, an irregular pattern has been observed in patients in older age groups [3, 6], which might be associated with various factors, such as the multiple comorbidities, physiological reserves, social/cognitive status, and the cancer stage at diagnosis [7]. Thus, an in-depth understanding of the associated underlying mechanisms could improve patient care and surgical outcomes.

According to current UK projections, by 2030, 76% of men and 70% of women with cancer will be over 65 years at the time of diagnosis [8]. It is known that older patients with neoplastic disease are often under-treated and under-represented in clinical trials, and they tend to have poorer surgical outcomes than their younger counterparts [9–12]; thus, chronological age alone is a poor predictor of cancer treatment tolerance [13]. Since diverse disease symptoms are observed in older cancer patients, an individualized, comprehensive preoperative assessment that would consider all comorbidities along with a multidisciplinary strategy would guarantee appropriate care and treatment for older CRC patients. A better understanding of the epidemiology of multimorbidity would help in designing more effective health care models to treat patients with multiple morbidities based on a benefit/risk assessment. This study aimed to evaluate the impact of age and multimorbidity on peri- and postoperative outcomes in patients who underwent surgical resection for CRC.

## Methods

### Study design and data source

Veneto is a region located in northeastern Italy with approximately 5 million inhabitants. The regional government is known to provide universal health care, which includes access to surgical units capable of performing any CRC surgical procedure. Only 3.2% of patients from Veneto region are operated out-of-region [14].

The primary information source of this study was the discharge dataset obtained from the regional hospitals. The dataset contained the following information: patient demographic data, admission and discharge dates of the patients, the primary and secondary diagnosis codes, the dates and codes corresponding to a maximum of six procedures performed during hospitalization (the International Classification of Diseases 9th Revision Clinical Modification 2007, ICD-9-CM), the American Society of Anesthesiologists (ASA) score [15], and whether the CRC procedure was urgent or elective. The Barthel index [16] was used to measure patients’ activities of daily living (ADL) at the time of admission. The study also gleaned information from death certificates, collected by the Local Health Units of the National Health Service, and transmitted to the Regional Epidemiology Service.

The study investigators also used the Johns Hopkins ACG^®^ System, which evaluates the multidimensional nature of a patient’s health. It is a population health system tool to facilitate person-focused healthcare management, i.e., it converts patient data from several sources into actionable information. The model, which has been validated in multiple European and non-European countries, is used to classify a regional population, providing several metrics that allow an accurate representation of the morbidity burden [17, 18]. The ACG metrics and algorithms were used in this study to identify patients affected by specific pathologies [19–21]. The record-linkage was performed on previously anonymized records to protect the privacy of all individuals involved in the study.

### Patient selection and classifications

The patients’ diseases were classified based on ICD-9-CM. Our study included all patients  ≥ 40 years who were admitted to any hospital in Veneto with a diagnosis of primary colon cancer (ICD9-CM 153.x) or rectal cancer (ICD9-CM 154.x) and who underwent urgent or elective surgical procedure between January 2013 and December 2015. The ICD9-CM procedure codes for CRC surgery were: 45.7x, 45.8, 48.35, 48.49, 48.5, 48.6x, and 45.95.

The exclusion criteria included: cancer of the anus (ICD9-CM 154.2, 154.3), patients who underwent surgical resection before January 1, 2013, patients who underwent ostomy surgery before index hospitalization (defined as the first of a series of hospitalizations) [14].

### Outcomes

The primary outcomes included: pre- and postoperative length of stay (LOS), 30-day readmission, 30-day reoperation and OS. The preoperative LOS was defined as the duration between the admission date and the date of surgical procedure; the postoperative LOS was defined as the duration between the date of surgical procedure and the date of discharge; 30-day readmission was defined as any unplanned hospitalization within 30 days of the date of discharge of the index hospitalization; 30-day reoperation was defined as any unplanned postoperative procedure, which involved an operating room or an imaging-guided intervention during 30 days following the index surgical procedure [14]; the OS was defined as death due to any cause after the surgical procedure; the monitoring follow-up for OS was 365 days. The pre- and postoperative LOS patient data were based on a cutoff of 4 and 8 days for pre- and post-hospitalization period, respectively (Table 3).

These outcomes are widely used to measure the quality of perioperative care since they can be easily retrieved from administrative databases.

### Age, surgical approach, and additional covariates

The age of the patients at the time of surgery was recorded. We defined four age classes (40–64, 65–74, 75–84, and 85+ years) in this analysis. The additional covariates used to assess and predict the outcomes of interest were as follows: open vs. laparoscopic approach, gender (male, female), Barthel Index Code (0–50: dependent state; 55–100: independent state), surgical complexity, and comorbidity indexes. The surgical complexity of the procedure was evaluated based on the following criteria: non-CRC surgical procedure-related hospitalizations during the year preceding the index hospitalization, hospitalizations for abdominal non-CRC-related surgery 3 years preceding index hospitalization, and stoma creation during the index hospitalization. We used the following two indexes to evaluate patient’s comorbidities: the Charlson Index [22], calculated for the 3 years preceding index hospitalization and a few ACG metrics to quantify the morbidity burden (i.e., the number of comorbidities) and to identify the primary chronic conditions affecting each patient [i.e., hypertension, lipid metabolism disorders, diabetes, osteoporosis, asthma, depression, glaucoma, congestive heart failure (CHF), hypothyroidism, chronic renal failure (CRF), chronic obstructive pulmonary disease (COPD), dementia, Parkinson’s disease, degenerative maculopathy, and rheumatoid arthritis (RA)]. The number of comorbidities was calculated based on the Expanded Diagnosis Clusters (EDCs) assigned to the patient by the ACG system. EDCs are diagnostic groupings (based on the patient’s diagnosis codes) that describe a pathology or related pathologies based on the organ or apparatus involved.

### Statistical analysis

The demographic and clinical characteristics of the patients were assessed using the *χ*^2^ test and the Cochran–Armitage’s trend test. The multivariable logistic regression analysis with stepwise backward selection was used to identify the significant predictors for each study outcome (mortality, readmission, LOS) (the significance level to remove an independent variable from the model was 0.10; the significance level to add an independent variable to the model was 0.05). The following variables were always included in the model: gender, admission modality (elective/urgent), number of hospitalizations preceding the index hospitalization, abdominal surgery during 3 years preceding index surgical procedure, stoma creation during the index hospitalization, the surgical site, and the type of surgical approach.

Multilevel regression was performed to account for the hierarchical structure of the data (first level: patient; second level: hospital). The overall survival was studied using Cox proportional hazards regression. The Cox regression assumptions were previously evaluated with global Schoenfeld’s test.

Statistical significance was set at *P* < 0.05. Stata software was used to perform all the analyses (Stata Corporation, Stata Statistical Software: Release 13.0. College Station, TX).

## Results

**Fig. 1:**
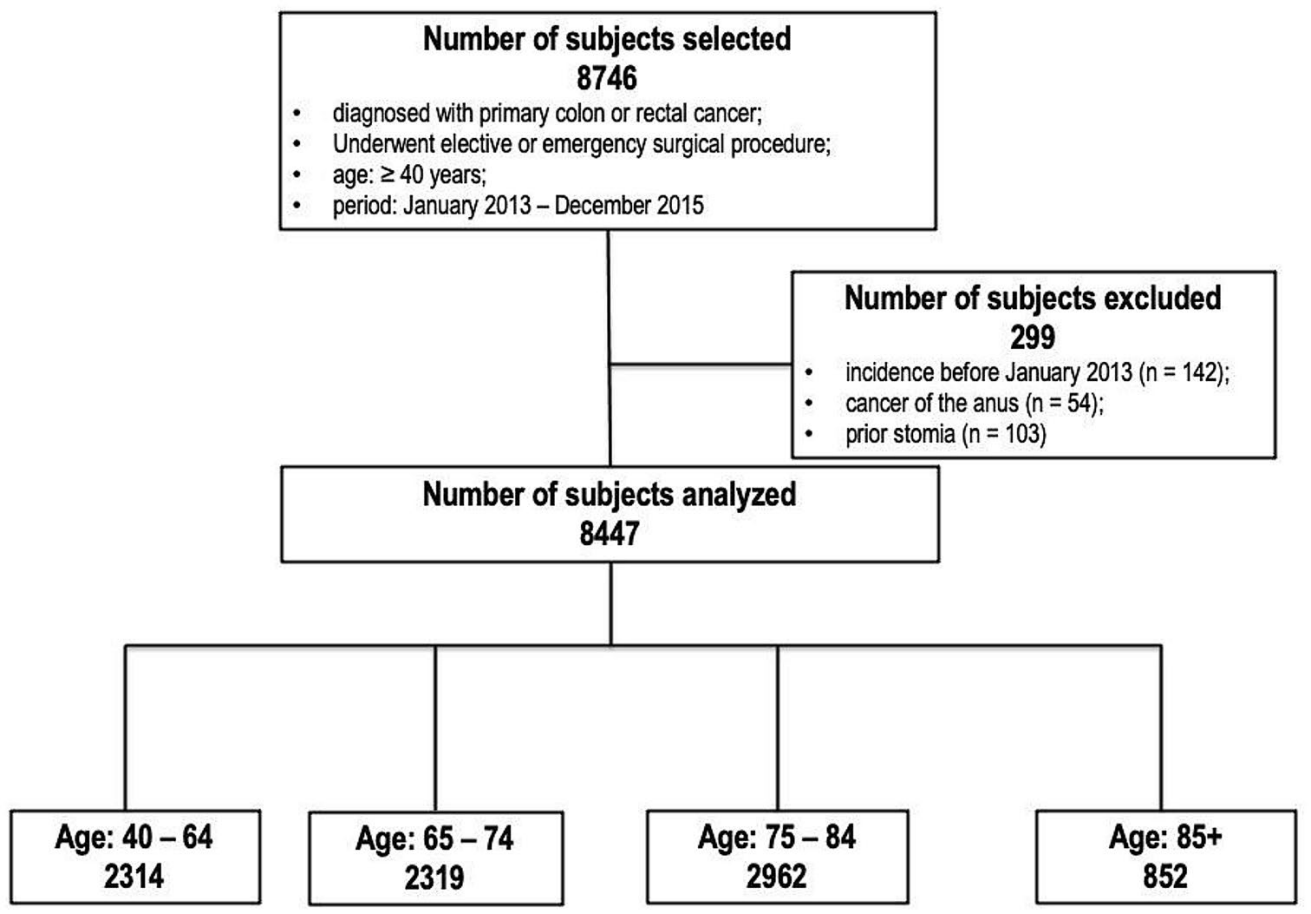
Study flow-chart

During the study period, 8447 Veneto residents ≥ 40 years underwent elective or urgent primary CRC resection. Figure 1 illustrates the flow-chart of the patients included in this study.

Table 1 outlines the patient characteristics. We found that 87.6% and 59.3% of the patients in the 40–64 year and 85+ year age classes underwent elective surgery (*P* < 0.01).

**Table 1:** Main characteristics of study patients by age class

	Total 8447 *n*	Age class				*P* value
		40–64 year 2314 *n* (%)	65–74 year 2319 *n* (%)	75–84 year 2962 *n* (%)	85+ year 852 *n* (%)	
Gender						
Male	4710	1281 (55.4)	1421 (61.3)	1652 (55.8)	356 (41.8)	< 0.01
Female	3737	1033 (40.6)	898 (38.7)	1310 (44.2)	496 (58.2)	
Admission modality						
Emergent/urgent	1656	286 (12.4)	333 (14.4)	690 (23.3)	347 (40.7)	< 0.01
Elective	6791	2028 (87.6)	1986 (85.6)	2272 (76.7)	505 (59.3)	
Hospitalization in the year before the index surgery						
None	6355	1894 (81.4)	1779 (76.5)	2126 (71.8)	556 (65.3)	< 0.01
One	1499	318 (13.7)	379 (16.3)	585 (19.8)	217 (25.5)	
More than one	593	102 (4.4)	161 (6.9)	251 (8.5)	79 (9.3)	
Abdominal surgery in the 3 years before the index surgery						
No	7938	2244 (97.0)	2222 (95.8)	2721 (91.9)	751 (88.1)	< 0.01
Yes	509	70 (3.0)	97 (4.2)	241 (8.1)	101 (11.9)	
Stoma creation in the index hospitalization						
No	7001	1873 (80.9)	1935 (83.4)	2458 (83.0)	735 (86.3)	< 0.01
Yes	1446	441 (19.1)	384 (16.6)	504 (17.0)	117 (13.7)	
Site						
Proximal	3789	878 (37.9)	1047 (45.1)	1404 (47.4)	460 (54.0)	< 0.01
Distal	4486	1403 (60.6)	1227 (52.9)	1481 (50.0)	375 (44.0)	
Other	172	33 (1.4)	45 (1.9)	77 (2.6)	17 (2.0)	
Charlson score						
None	6915	2141 (92.5)	1927 (83.1)	2234 (75.4)	613 (71.9)	< 0.01
1–2	1317	151 (6.5)	354 (15.3)	619 (20.9)	193 (22.7)	
3+	215	22 (1.0)	38 (1.6)	109 (3.7)	46 (5.4)	
Number of comorbidities*						
1	2269	1108 (47.9)	571 (24.6)	490 (16.5)	100 (4.3)	< 0.01
2	2311	681 (29.4)	700 (30.2)	753 (25.4)	177 (20.8)	
3	1624	303 (13.1)	469 (20.2)	643 (21.7)	209 (24.5)	
4	1035	130 (5.6)	275 (11.9)	472 (15.9)	158 (18.5)	
5+	1209	92 (4.0)	304 (13.1)	604 (20.4)	209 (24.5)	
Barthel index at the time of admission						
Dependent (0–50)	1425	182 (7.9)	308 (13.3)	593 (20.0)	342 (40.2)	< 0.01
Independent (55–100)	5819	1736 (75.0)	1700 (73.3)	1983 (66.9)	400 (46.9)	
Missing	1203	396 (17.1)	311 (13.4)	386 (13.0)	110 (12.9)	
ASA Score						
ASA 1 or 2	4373	1587 (68.6)	1339 (57.7)	1260 (42.5)	187 (21.9)	< 0.01
ASA 3	2391	272 (11.6)	549 (23.4)	1106 (36.7)	464 (52.9)	
ASA 4+	209	13 (0.7)	39 (2.0)	89 (3.6)	68 (9.5)	
Missing	1474	442 (19.1)	392 (16.9)	507 (17.1)	133 (15.6)	
Surgical approach						
Open	4675	1034 (44.7)	1237 (53.3)	1760 (59.4)	644 (75.6)	< 0.01
Laparoscopic	3772	1280 (55.3)	1082 (46.7)	1202 (40.6)	208 (24.4)	
Modality of discharge						
Death	180	11 (0.5)	26 (1.1)	89 (3.0)	54 (6.3)	< 0.01
Home	7982	2290 (98.6)	2229 (96.1)	2723 (91.9)	708 (83.1)	
Non-home	285	13 (0.9)	64 (2.8)	150 (5.1)	90 (10.6)	

*CRC is included in the number of comorbidities

We found three or more comorbidities in 22.7% of patients aged 40–64 years and 67.5% in patients ≥ 85 years (Tab. 1). Based on the Barthel Index at hospital admission, the younger patients showed more independence in carrying out ADL, and higher (worse) values were associated with increasing age; the following patients (%) scored between 0 and 50: 7.9%, 13.3%, 20.0%, and 40.2% aged 40–64 years, 65–74 years, 75–84 years, and 85+ years, respectively (*P* < 0.01) (Table 1).

During the study period, the laparoscopic approach was used more frequently in the younger patients than in the older patients: 55.3% in the 40–64 years age class, and 24.4% in the 85+ years age class (*P* < 0.01) (Table 1).

Most patients (7982) were directly discharged to home (Table 1). There was an age-related correlation regarding discharge to non-home settings (i.e., intermediate care or nursing home), with the highest values in the oldest groups (0.9% in 40–64 years class vs. 10.6% in 85+ years; *P* < 0.001) (Table 1).

The most frequent comorbidities based on the ACG system were hypertension (63.7%), followed by lipid metabolism disorders (28.2%), diabetes (17.7%), osteoporosis (16.6%), anemia (14.6%), asthma/COPD (13.6%), depression (8.9%), glaucoma (5.4%), CHF (4.8%), hypothyroidism (4.7%), and CRF (3.2%). There was an age-related increase in the prevalence of these conditions (*P* < 0.01) (Table 2).

**Table 2:** Comorbidity^a^ cohort

	Total 8447 *n* (%)	Age class			
		40–64 years 2314 *n* (%)	65–74 years 2919 *n* (%)	75–84 years 2962 *n* (%)	85+ years 852 *n* (%)
Pathologies					
Hypertension	5386 (63.7)	885 (38.2)	1508 (65.0)	2308 (77.9)	685 (80.4)
Disorders of lipid metabolism	2382 (28.2)	332 (14.3)	732 (31.6)	1088 (36.7)	230 (27.0)
Diabetes	1497 (17.7)	213 (9.2)	496 (21.4)	629 (21.2)	159 (18.7)
Osteoporosis	1408 (16.6)	249 (10.8)	389 (16.8)	573 (19.3)	197 (23.1)
Anemia*	1237 (14.6)	193 (8.3)	262 (11.3)	539 (18.2)	243 (28.5)
Asthma/COPD^Ɨ^	1152 (13.6)	228 (9.9)	303 (13.1)	447 (15.1)	174 (20.4)
Depression	759 (8.9)	139 (6.0)	188 (8.1)	322 (10.9)	110 (12.9)
Glaucoma	462 (5.4)	46 (2.0)	114 (4.9)	236 (8.0)	66 (7.7)
Congestive heart failure (CHF)	408 (4.8)	18 (0.8)	85 (3.7)	197 (6.6)	110 (12.9)
Hypothyroidism	398 (4.7)	97 (4.2)	111 (4.8)	131 (4.4)	59 (6.9)
Chronic renal failure (CRF)	269 (3.2)	24 (1.0)	63 (2.7)	132 (4.5)	50 (5.9)
Dementia	239 (2.8)	8 (0.3)	32 (1.4)	128 (4.3)	71 (8.3)
Parkinson’s disease	154 (1.8)	12 (0.5)	39 (1.7)	76 (2.6)	27 (3.2)
Degenerative maculopathy	134 (1.6)	55 (2.4)	29 (1.3)	41 (1.4)	9 (1.1)
Rheumatoid arthritis	101 (1.2)	14 (0.6)	25 (1.1)	49 (1.7)	13 (1.5)

^a^Comorbidity identification performed according to the ACG system algorithms

^b^The Cochran–Armitage test for trend performed for each pathology resulted always significant (*p* < 0.001)

*Iron deficiency, other deficiency anemias

^Ɨ^Chronic obstructive pulmonary disease

Only 15.9% of the patients waited longer than 4 days for the surgical procedure during the hospitalization (Table 3). The preoperative LOS showed an age-related increase and was longer in patients undergoing urgent procedures: 58.5% of the patients undergoing urgent procedures waited more than 4 days for the surgical procedure compared with 5.5% of the elective patients (*P* < 0.01).

**Table 3:** Outcome measures by age class

	Total (%)	Age class				*P* value
		40–64 years (%)	65–74 years (%)	75–84 years (%)	85+ years (%)	
Preoperative LOS ≥ 4 days*	15.9	9.4	12.3	19.6	30.9	< 0.001
Postoperative LOS ≥ 8 days*	49.5	37.6	47.5	55.7	65.9	< 0.001
30-day reoperation	7.1	7.2	6.6	6.8	5.4	0.681
30-day readmission	5.5	4.6	5.9	5.9	5.6	0.045
365-day mortality	11.7	5.1	8.5	15.0	27.1	< 0.001

*LOS length of stay, *Preoperative LOS* percentage of patients who had waited at least 4 days before surgery, *postoperative LOS* percentage of patients who had waited at least 8 days before discharge after surgery

Age was found to be related to all outcome measures, excluding the 30-day reoperation; we noted a minor significance for 30-day readmission (Table 3).

Adjusted ORs (Table 4) describe the association between age and outcome measures. While age represented a risk factor for longer preoperative and postoperative LOS as well as for overall survival (HR), it was not associated with an increased risk of 30-day reoperation and 30-day readmission.

**Table 4:** Association between outcome measures, age and comorbidities: adjusted odds ratio and hazard ratio with estimated 95% confidence interval

	Preoperative LOS (OR)	Postoperative LOS (OR)	30-day reoperation (OR)	30-day readmission (OR)	Overall survival (HR)
65–74	1.14 (0.91**–**1.11)	**1.49 (1.31–1.69)**	0.88 (0.69–1.12)	1.17 (0.89**–**1.53)	**1.50 (1.18–1.89)**
75–84	**1.42 (1.215–1.76)**	**1.86 (1.65–2.11)**	0.92 (0.73–1.16)	1.10 (0.85**–**1.43)	**2.22 (1.80–2.75)**
85+	**1.43 (1.09–1.86)**	**2.51 (2.09–3.01)**	0.85 (0.59–1.24)	1.03 (0.70**–**1.51)	**3.36 (2.64–4.26)**
Depression	–	**1.21 (1.02–1.42)**	–	–	**1.44 (1.12–1.86)**
Diabetes	**1.33 (1.10–1.61)**	–	0.78 (0.60**–**1.01)	**1.31 (1.03–1.66)**	–
CHF	**1.51 (1.13–2.02)**	**1.31 (1.05–1.65)**	–	–	**1.99 (1.63–2.42)**
Hypertension	**1.19 (1.01–1.41)**	1.10 (0.99–1.22)	–	–	–
Asthma/COPD	–	**1.18 (1.03–1.35)**	–	–	**1.39 (1.08–1.78)**
Parkinson disease	1.52 (0.94–2.45)	**1.52 (1.07–2.17)**	–	–	**–**
Dementia	–	–	–	**1.88 (1.19–2.95)**	**1.44 (1.12–1.86)**
Hypothyroidism	–	–	–	**1.52 (1.02–2.28)**	–
CRF	–	–	–	**1.82 (1.20–2.75)**	**1.45 (1.13–1.88)**
Anemia*	**2.34 (1.94–2.83)**	1.14 (0.99–1.31)	–	–	–

Values in bold indicate statistically significant results

Adjusted OR is calculated with multivariable logistic regression analysis with stepwise backward selection. Independent variables: gender, age, admission modality, hospitalization in the year before the index surgery, abdominal surgery 3 years before the index surgery, stoma creation in the index hospitalization, site surgery, surgical approach and comorbidities (ACG). Adjusted OR was not reported for the diseases excluded by stepwise backward selection

Adjusted HR is calculated with Cox regression model. Independent variables: gender, age, admission modality, hospitalization in the year before the index surgery, abdominal surgery 3 years before the index surgery, stoma creation in the index hospitalization, site surgery, surgical approach and comorbidities (ACG)

Adjusted OR and HR was not reported for the diseases excluded by stepwise backward selection

*LOS* length of stay; * (iron deficiency, other deficiency anemias)

Adjusted ORs were also calculated to examine the association between comorbidities and the outcome measures. CHF was associated with longer preoperative (OR 1.51, 95% CI 1.13–2.02) and postoperative (OR 1.31, 95% CI 1.05–1.65) LOS as well as with the OS (HR 1.99, 95% CI 1.63–2.42). CRF was associated with 30-day readmission (OR 1.82, 95% CI 1.20–2.75) as well as with the OS (HR 1.45, 95% CI 1.13–1.88). Parkinson’s disease was associated with postoperative LOS (OR 1.52, 95% CI 1.07–2.17). Depression was associated with postoperative LOS (OR 1.21, 95% CI 1.02–1.42) and OS (HR 1.44, 95% CI 1.12–1.86). Dementia was associated with 30-day readmission (OR 1.88, 95% CI 1.19–2.95) and OS (HR 1.44, 95% CI 1.12–1.86). Anemia was associated exclusively with preoperative LOS (OR 2.34, 95% CI 1.94–2.83) (Table 4).

## Discussion

Several studies have reported that advanced age and pre-existing cardiac pathology are risk factors for postoperative complications in older patients with CRC [23–25]. Our data showed that age did predict longer pre- and postoperative LOS, as well as OS. However, in the study cohort, age did not affect 30-day reoperation and 30-day postoperative readmission rates.

Older patients are known to be more likely to develop postoperative complications in emergencies compared with their younger counterparts. In fact, older patients who undergo emergency procedures have 3–10 times higher rate of morbidity and mortality than those who undergo elective surgery [26].

Our data analysis did not find any association between old age and 30-day reoperation and 30-day postoperative readmission rates, confirming that chronologic age itself did not negatively influence surgical outcomes in CRC patients. Moreover, after adjusting for age, we found a significant and independent association between Diabetes, Dementia, Hypothyroidism, CRF, and 30-day postoperative readmission. Depression, CHF, Asthma/COPD, and Dementia significantly predicted OS in our patients, and along with CRF.

Since multimorbidity indicates a more vulnerable health status that increases the risk of adverse outcomes [27], identifying comorbidities is an important step in managing cancer patients that require surgical resection. Both surgical and systemic oncological treatments are stressors that can potentially challenge the physiological reserves of an older patient [28]. Moreover, older patients with CRC are characterized by lower cancer-related survival rates, which might be attributed to less aggressive treatment [29].

Lemmens et al. conducted a comprehensive assessment in older patients undergoing surgical tumor resection at the time of diagnosis to uncover existing comorbid conditions that could predict complications and/or less favorable outcomes and mortality [30]. They found that the assessment helped to facilitate surgical planning and to offer the best therapeutic options to the patients and caregivers. Furthermore, they stated that the use of pre-habilitation programs, especially in patients undergoing planned surgical procedures, could help improve the preoperative physical condition and could result in improved cancer outcome [30].

Another study conducted a multidisciplinary preoperative assessment to reveal known risk factors for postoperative complications that could be improved in the preoperative period, such as poly-medication, malnutrition, dehydration, and electrolyte imbalance [31] with the intent to reduce the older individuals’ preoperative LOS and postoperative complications, such as delirium [32].

In older patients undergoing surgical tumor resection, the implementation of pre-habilitation programs, fast track protocols, or ERAS bundles can have positive effects on the outcomes considered in the present study [33, 34].

While this study included a large sample size and confirmed data on patients’ comorbidities from administrative databases, it had several limitations, including the lack of information on the cancer stage, chemotherapy/radiotherapy treatments, the severity of comorbidities (CHF-NYHA class) in patients, and the distribution of some important risk factors and their management.

## Conclusions and implications

Data analysis confirmed that age affected the short- and long-term outcomes in this study cohort of older patients that required surgical treatment for CRC. A comprehensive pre-surgical assessment of the patients’ comorbidities could facilitate the prognostic analysis and help to calculate the risk–benefit ratio as well as to select the best therapeutic option for the CRC patient with multimorbidity. Future studies need to consider the patients’ cancer stage, the severity of comorbidities, and other risk factors to evaluate other dimensions of disease complexity.
